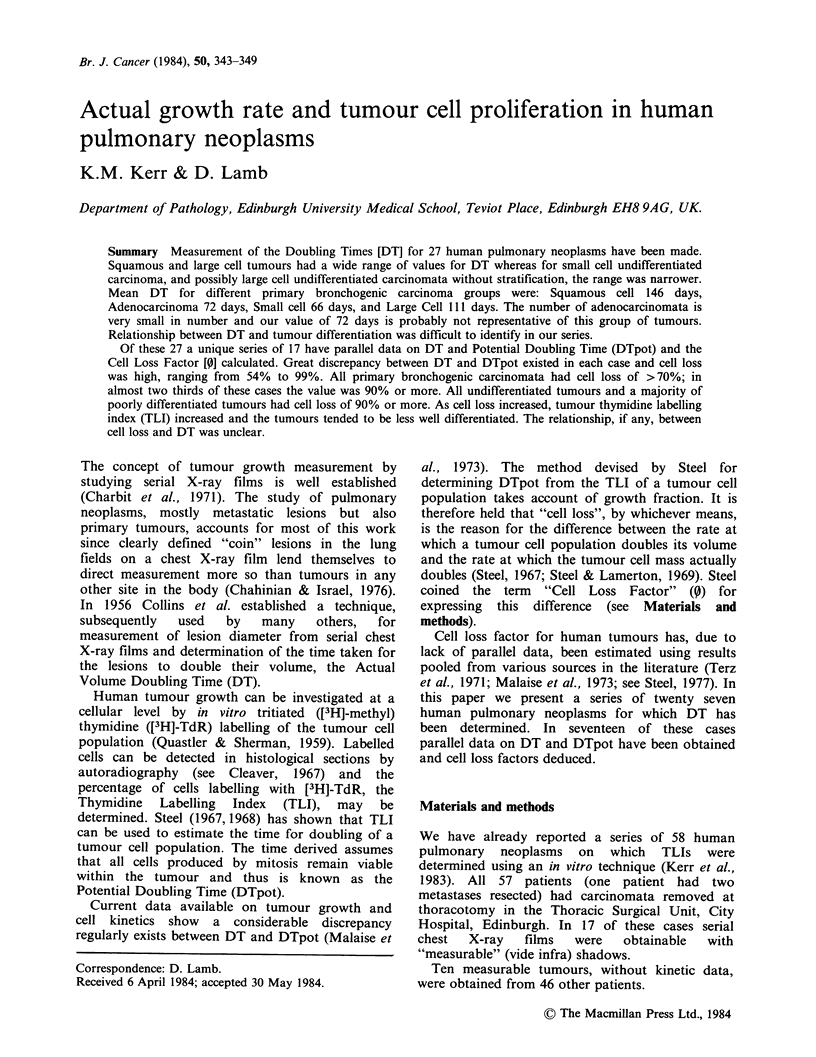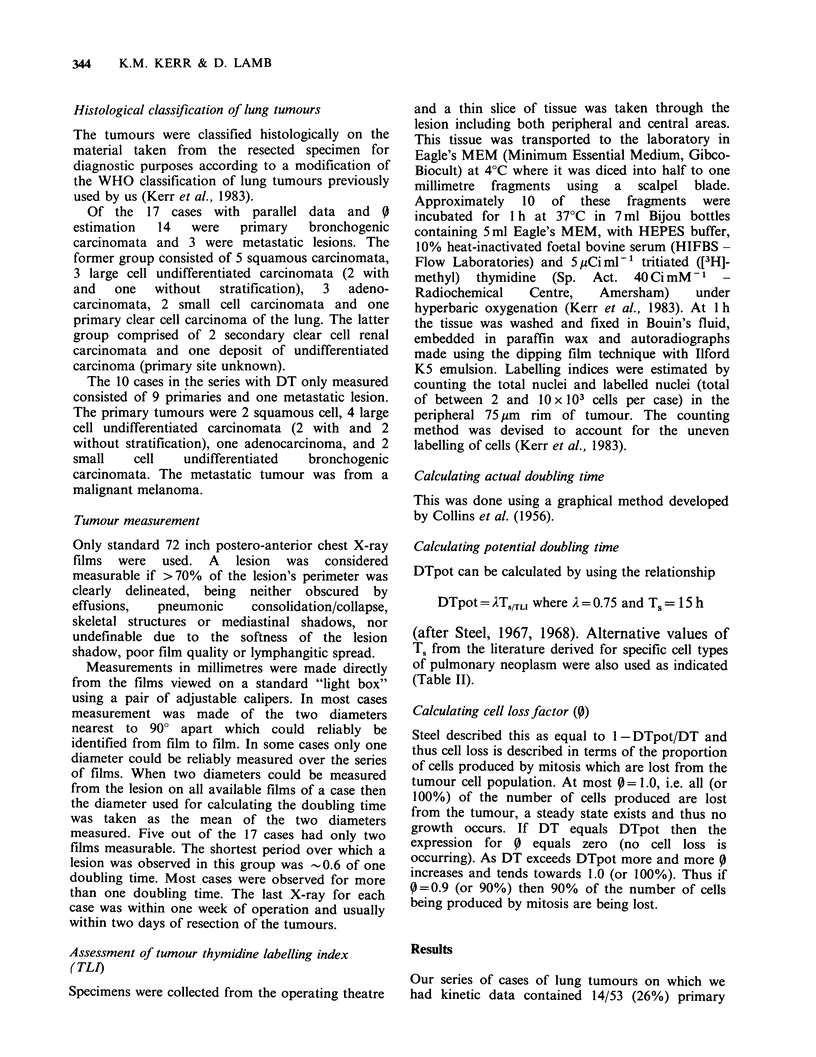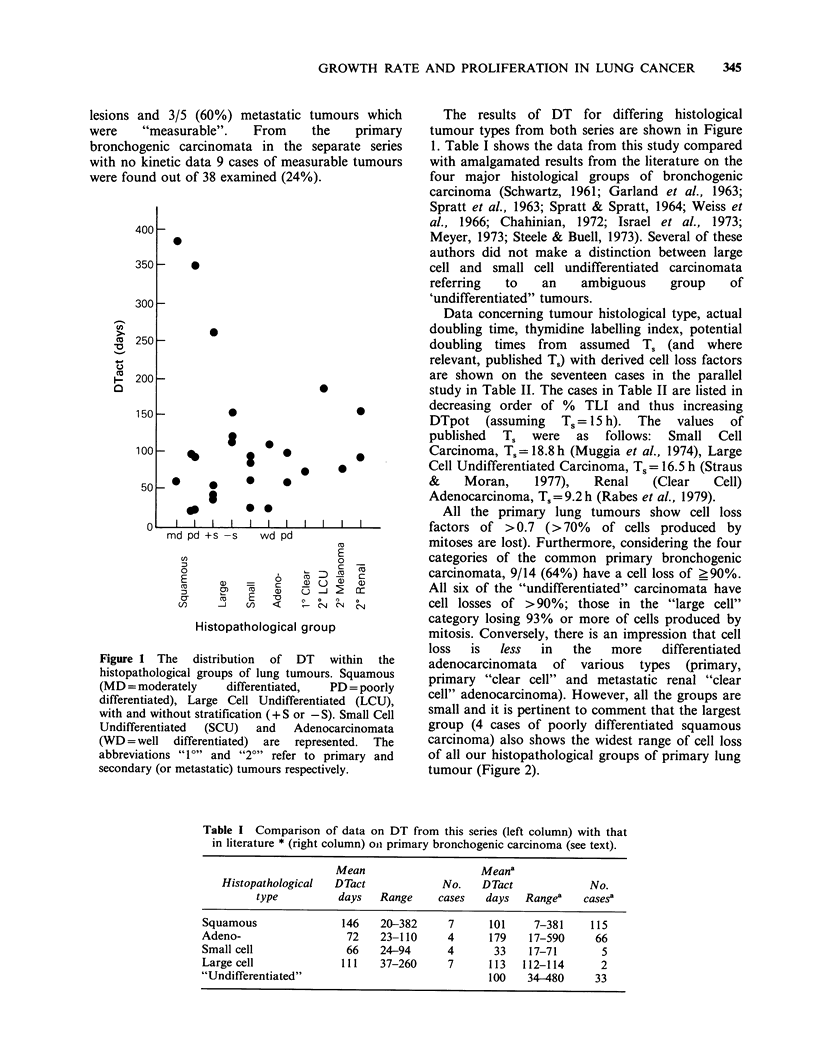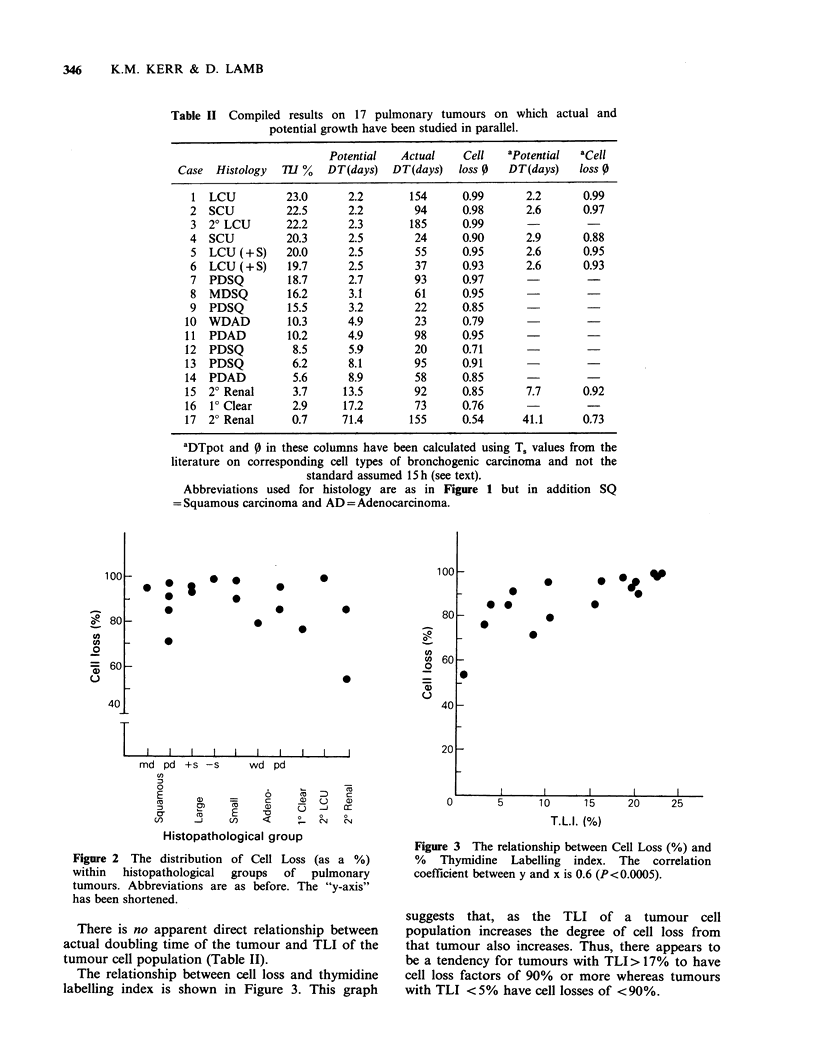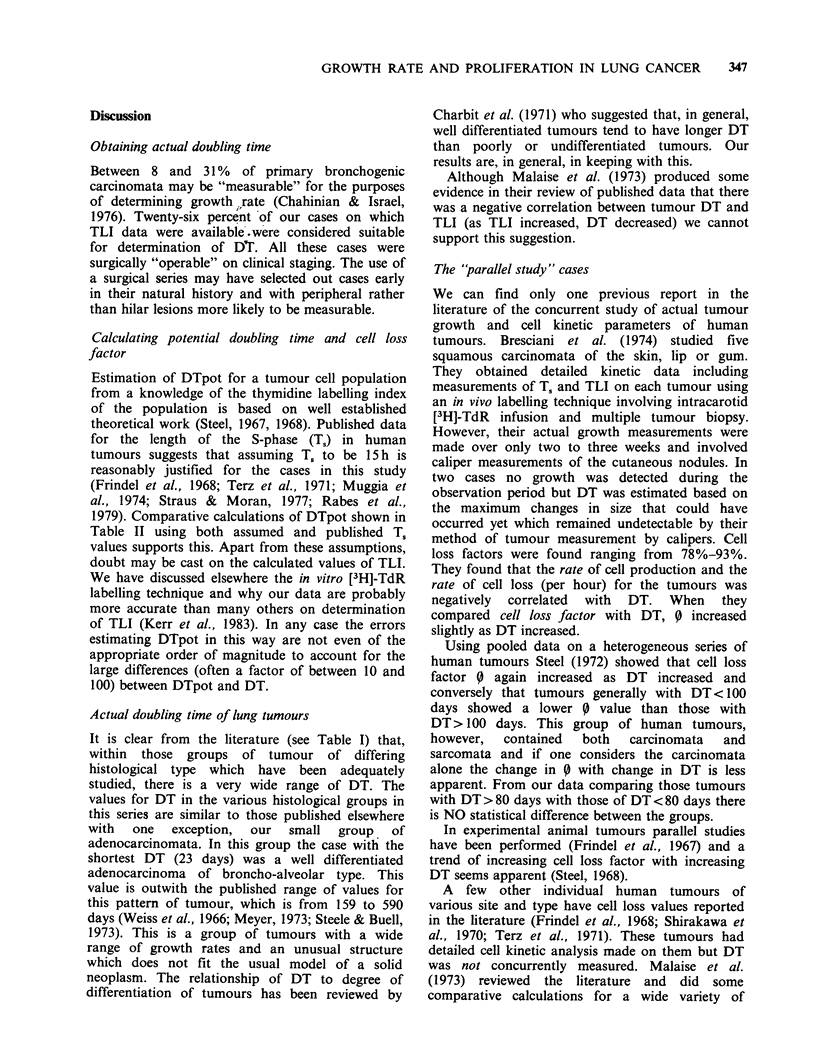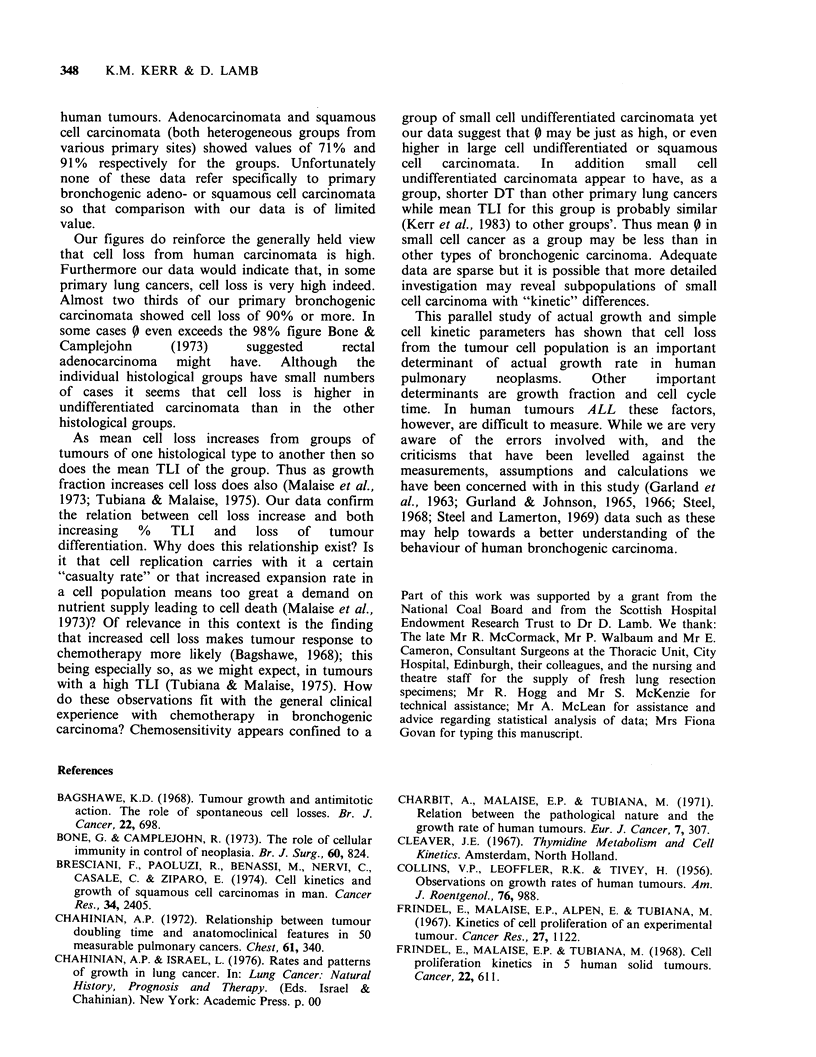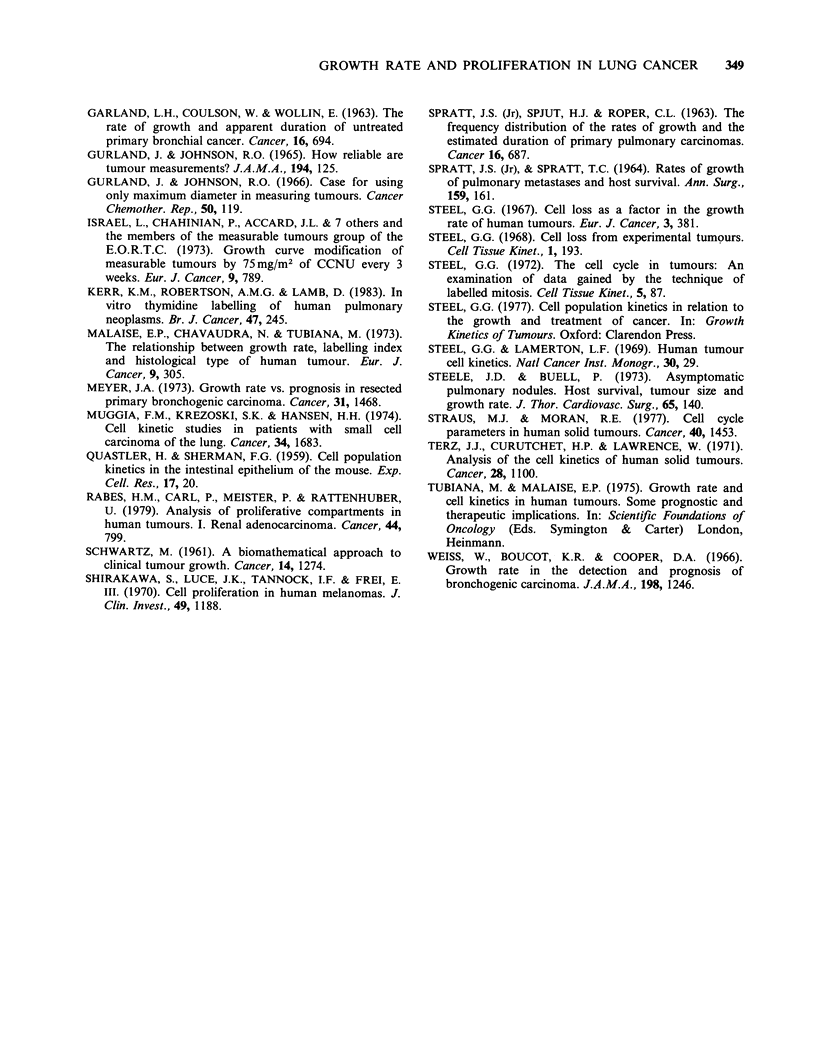# Actual growth rate and tumour cell proliferation in human pulmonary neoplasms.

**DOI:** 10.1038/bjc.1984.181

**Published:** 1984-09

**Authors:** K. M. Kerr, D. Lamb

## Abstract

Measurement of the Doubling Times [DT] for 27 human pulmonary neoplasms have been made. Squamous and large cell tumours had a wide range of values for DT whereas for small cell undifferentiated carcinoma, and possibly large cell undifferentiated carcinomata without stratification, the range was narrower. Mean DT for different primary bronchogenic carcinoma groups were: Squamous cell 146 days, Adenocarcinoma 72 days, Small cell 66 days, and Large Cell 111 days. The number of adenocarcinomata is very small in number and our value of 72 days is probably not representative of this group of tumours. Relationship between DT and tumour differentiation was difficult to identify in our series. Of these 27 a unique series of 17 have parallel data on DT and Potential Doubling Time (DTpot) and the Cell Loss Factor [0] calculated. Great discrepancy between DT and DTpot existed in each case and cell loss was high, ranging from 54% to 99%. All primary bronchogenic carcinomata had cell loss of greater than 70%; in almost two thirds of these cases the value was 90% or more. All undifferentiated tumours and a majority of poorly differentiated tumours had cell loss of 90% or more. As cell loss increased, tumour thymidine labelling index (TLI) increased and the tumours tended to be less well differentiated. The relationship, if any, between cell loss and DT was unclear.


*Br. J. Cancer* (1984), **50**, 343–349

### Actual growth rate and tumour cell proliferation in human pulmonary neoplasms

K.M. Kerr & D. Lamb

*Department of Pathology, Edinburgh University Medical School, Teviot Place, Edinburgh EH8 9AG, UK.*

**Summary** Measurement of the Doubling Times [DT] for 27 human pulmonary neoplasms have been made. Squamous and large cell tumours had a wide range of values for DT whereas for small cell undifferentiated carcinoma, and possibly large cell undifferentiated carcinomata without stratification, the range was narrower. Mean DT for different primary bronchogenic carcinoma groups were: Squamous cell 146 days, Adenocarcinoma 72 days, Small cell 66 days, and Large Cell 111 days. The number of adenocarcinomata is very small in number and our value of 72 days is probably not representative of this group of tumours. Relationship between DT and tumour differentiation was difficult to identify in our series.

Of these 27 a unique series of 17 have parallel data on DT and Potential Doubling Time (DTpot) and the Cell Loss Factor [∅] calculated. Great discrepancy between DT and DTpot existed in each case and cell loss was high, ranging from 54% to 99%. All primary bronchogenic carcinomata had cell loss of >70%; in almost two thirds of these cases the value was 90% or more. All undifferentiated tumours and a majority of poorly differentiated tumours had cell loss of 90% or more. As cell loss increased, tumour thymidine labelling index (TLI) increased and the tumours tended to be less well differentiated. The relationship, if any, between cell loss and DT was unclear.

The concept of tumour growth measurement by studying serial X-ray films is well established (Charbit *et al.*, 1971). The study of pulmonary neoplasms, mostly metastatic lesions but also primary tumours, accounts for most of this work since clearly defined "coin" lesions in the lung fields on a chest X-ray film lend themselves to direct measurement more so than tumours in any other site in the body (Chahinian & Israel, 1976). In 1956 Collins *et al.* established a technique, subsequently used by many others, for measurement of lesion diameter from serial chest X-ray films and determination of the time taken for the lesions to double their volume, the Actual Volume Doubling Time (DT).

Human tumour growth can be investigated at a cellular level by *in vitro* tritiated ([$^3$H]-methyl) thymidine ([$^3$H]-TdR) labelling of the tumour cell population (Quastler & Sherman, 1959). Labelled cells can be detected in histological sections by autoradiography (see Cleaver, 1967) and the percentage of cells labelling with [$^3$H]-TdR, the Thymidine Labelling Index (TLI), may be determined. Steel (1967, 1968) has shown that TLI can be used to estimate the time for doubling of a tumour cell population. The time derived assumes that all cells produced by mitosis remain viable within the tumour and thus is known as the Potential Doubling Time (DTpot).

Current data available on tumour growth and cell kinetics show a considerable discrepancy regularly exists between DT and DTpot (Malaise *et al.*, 1973). The method devised by Steel for determining DTpot from the TLI of a tumour cell population takes account of growth fraction. It is therefore held that "cell loss", by whichever means, is the reason for the difference between the rate at which a tumour cell population doubles its volume and the rate at which the tumour cell mass actually doubles (Steel, 1967; Steel & Lamerton, 1969). Steel coined the term "Cell Loss Factor" (∅) for expressing this difference (see **Materials and methods**).

Cell loss factor for human tumours has, due to lack of parallel data, been estimated using results pooled from various sources in the literature (Terz *et al.*, 1971; Malaise *et al.*, 1973; see Steel, 1977). In this paper we present a series of twenty seven human pulmonary neoplasms for which DT has been determined. In seventeen of these cases parallel data on DT and DTpot have been obtained and cell loss factors deduced.

### Materials and methods

We have already reported a series of 58 human pulmonary neoplasms on which TLIs were determined using an *in vitro* technique (Kerr *et al.*, 1983). All 57 patients (one patient had two metastases resected) had carcinomata removed at thoracotomy in the Thoracic Surgical Unit, City Hospital, Edinburgh. In 17 of these cases serial chest X-ray films were obtainable with "measurable" (vide infra) shadows.

Ten measurable tumours, without kinetic data, were obtained from 46 other patients.

Correspondence: D. Lamb.
Received 6 April 1984; accepted 30 May 1984.

© The Macmillan Press Ltd., 1984

*Histological classification of lung tumours*

The tumours were classified histologically on the material taken from the resected specimen for diagnostic purposes according to a modification of the WHO classification of lung tumours previously used by us (Kerr *et al.*, 1983).

Of the 17 cases with parallel data and Ø estimation 14 were primary bronchogenic carcinomata and 3 were metastatic lesions. The former group consisted of 5 squamous carcinomata, 3 large cell undifferentiated carcinomata (2 with and one without stratification), 3 adenocarcinomata, 2 small cell carcinomata and one primary clear cell carcinoma of the lung. The latter group comprised of 2 secondary clear cell renal carcinomata and one deposit of undifferentiated carcinoma (primary site unknown).

The 10 cases in the series with DT only measured consisted of 9 primaries and one metastatic lesion. The primary tumours were 2 squamous cell, 4 large cell undifferentiated carcinomata (2 with and 2 without stratification), one adenocarcinoma, and 2 small cell undifferentiated bronchogenic carcinomata. The metastatic tumour was from a malignant melanoma.

*Tumour measurement*

Only standard 72 inch postero-anterior chest X-ray films were used. A lesion was considered measurable if >70% of the lesion's perimeter was clearly delineated, being neither obscured by effusions, pneumonic consolidation/collapse, skeletal structures or mediastinal shadows, nor undefinable due to the softness of the lesion shadow, poor film quality or lymphangitic spread.

Measurements in millimetres were made directly from the films viewed on a standard "light box" using a pair of adjustable calipers. In most cases measurement was made of the two diameters nearest to 90° apart which could reliably be identified from film to film. In some cases only one diameter could be reliably measured over the series of films. When two diameters could be measured from the lesion on all available films of a case then the diameter used for calculating the doubling time was taken as the mean of the two diameters measured. Five out of the 17 cases had only two films measurable. The shortest period over which a lesion was observed in this group was ~0.6 of one doubling time. Most cases were observed for more than one doubling time. The last X-ray for each case was within one week of operation and usually within two days of resection of the tumours.

*Assessment of tumour thymidine labelling index (TLI)*

Specimens were collected from the operating theatre and a thin slice of tissue was taken through the lesion including both peripheral and central areas. This tissue was transported to the laboratory in Eagle's MEM (Minimum Essential Medium, Gibco-Biocult) at 4°C where it was diced into half to one millimetre fragments using a scalpel blade. Approximately 10 of these fragments were incubated for 1 h at 37°C in 7 ml Bijou bottles containing 5 ml Eagle's MEM, with HEPES buffer, 10% heat-inactivated foetal bovine serum (HIFBS – Flow Laboratories) and 5 $\mu$Ci ml$^{-1}$ tritiated ([$^3$H]-methyl) thymidine (Sp. Act. 40 Ci mM$^{-1}$ – Radiochemical Centre, Amersham) under hyperbaric oxygenation (Kerr *et al.*, 1983). At 1 h the tissue was washed and fixed in Bouin's fluid, embedded in paraffin wax and autoradiographs made using the dipping film technique with Ilford K5 emulsion. Labelling indices were estimated by counting the total nuclei and labelled nuclei (total of between 2 and $10 \times 10^3$ cells per case) in the peripheral 75 $\mu$m rim of tumour. The counting method was devised to account for the uneven labelling of cells (Kerr *et al.*, 1983).

*Calculating actual doubling time*

This was done using a graphical method developed by Collins *et al.* (1956).

*Calculating potential doubling time*

DTpot can be calculated by using the relationship

$$\mathrm{DTpot} = \lambda T_{s/TLI} \text{ where } \lambda = 0.75 \text{ and } T_s = 15\,\mathrm{h}$$

(after Steel, 1967, 1968). Alternative values of $T_s$ from the literature derived for specific cell types of pulmonary neoplasm were also used as indicated (Table II).

*Calculating cell loss factor* (Ø)

Steel described this as equal to 1 – DTpot/DT and thus cell loss is described in terms of the proportion of cells produced by mitosis which are lost from the tumour cell population. At most Ø = 1.0, i.e. all (or 100%) of the number of cells produced are lost from the tumour, a steady state exists and thus no growth occurs. If DT equals DTpot then the expression for Ø equals zero (no cell loss is occurring). As DT exceeds DTpot more and more Ø increases and tends towards 1.0 (or 100%). Thus if Ø = 0.9 (or 90%) then 90% of the number of cells being produced by mitosis are being lost.

### Results

Our series of cases of lung tumours on which we had kinetic data contained 14/53 (26%) primary lesions and 3/5 (60%) metastatic tumours which were "measurable". From the primary bronchogenic carcinomata in the separate series with no kinetic data 9 cases of measurable tumours were found out of 38 examined (24%).

**Figure 1** The distribution of DT within the histopathological groups of lung tumours. Squamous (MD = moderately differentiated, PD = poorly differentiated), Large Cell Undifferentiated (LCU), with and without stratification (+S or −S). Small Cell Undifferentiated (SCU) and Adenocarcinomata (WD = well differentiated) are represented. The abbreviations "1°" and "2°" refer to primary and secondary (or metastatic) tumours respectively.

The results of DT for differing histological tumour types from both series are shown in Figure 1. Table I shows the data from this study compared with amalgamated results from the literature on the four major histological groups of bronchogenic carcinoma (Schwartz, 1961; Garland *et al.*, 1963; Spratt *et al.*, 1963; Spratt & Spratt, 1964; Weiss *et al.*, 1966; Chahinian, 1972; Israel *et al.*, 1973; Meyer, 1973; Steele & Buell, 1973). Several of these authors did not make a distinction between large cell and small cell undifferentiated carcinomata referring to an ambiguous group of 'undifferentiated" tumours.

Data concerning tumour histological type, actual doubling time, thymidine labelling index, potential doubling times from assumed $T_s$ (and where relevant, published $T_s$) with derived cell loss factors are shown on the seventeen cases in the parallel study in Table II. The cases in Table II are listed in decreasing order of % TLI and thus increasing DTpot (assuming $T_s = 15\,h$). The values of published $T_s$ were as follows: Small Cell Carcinoma, $T_s = 18.8\,h$ (Muggia *et al.*, 1974), Large Cell Undifferentiated Carcinoma, $T_s = 16.5\,h$ (Straus & Moran, 1977), Renal (Clear Cell) Adenocarcinoma, $T_s = 9.2\,h$ (Rabes *et al.*, 1979).

All the primary lung tumours show cell loss factors of >0.7 (>70% of cells produced by mitoses are lost). Furthermore, considering the four categories of the common primary bronchogenic carcinomata, 9/14 (64%) have a cell loss of ≧90%. All six of the "undifferentiated" carcinomata have cell losses of >90%; those in the "large cell" category losing 93% or more of cells produced by mitosis. Conversely, there is an impression that cell loss is *less* in the more differentiated adenocarcinomata of various types (primary, primary "clear cell" and metastatic renal "clear cell" adenocarcinoma). However, all the groups are small and it is pertinent to comment that the largest group (4 cases of poorly differentiated squamous carcinoma) also shows the widest range of cell loss of all our histopathological groups of primary lung tumour (Figure 2).

**Table I** Comparison of data on DT from this series (left column) with that in literature * (right column) on primary bronchogenic carcinoma (see text).

| *Histopathological type* | *Mean DTact days* | *Range* | *No. cases* | *Mean[a] DTact days* | *Range[a]* | *No. cases[a]* |
|---|---|---|---|---|---|---|
| Squamous | 146 | 20–382 | 7 | 101 | 7-381 | 115 |
| Adeno- | 72 | 23–110 | 4 | 179 | 17-590 | 66 |
| Small cell | 66 | 24–94 | 4 | 33 | 17–71 | 5 |
| Large cell | 111 | 37-260 | 7 | 113 | 112-114 | 2 |
| "Undifferentiated" | | | | 100 | 34–480 | 33 |

**Table II** Compiled results on 17 pulmonary tumours on which actual and potential growth have been studied in parallel.

| *Case* | *Histology* | *TLI %* | *Potential DT(days)* | *Actual DT(days)* | *Cell loss ∅* | [a]*Potential DT(days)* | [a]*Cell loss ∅* |
|---|---|---|---|---|---|---|---|
| 1 | LCU | 23.0 | 2.2 | 154 | 0.99 | 2.2 | 0.99 |
| 2 | SCU | 22.5 | 2.2 | 94 | 0.98 | 2.6 | 0.97 |
| 3 | 2° LCU | 22.2 | 2.3 | 185 | 0.99 | — | — |
| 4 | SCU | 20.3 | 2.5 | 24 | 0.90 | 2.9 | 0.88 |
| 5 | LCU (+S) | 20.0 | 2.5 | 55 | 0.95 | 2.6 | 0.95 |
| 6 | LCU (+S) | 19.7 | 2.5 | 37 | 0.93 | 2.6 | 0.93 |
| 7 | PDSQ | 18.7 | 2.7 | 93 | 0.97 | — | — |
| 8 | MDSQ | 16.2 | 3.1 | 61 | 0.95 | — | — |
| 9 | PDSQ | 15.5 | 3.2 | 22 | 0.85 | — | — |
| 10 | WDAD | 10.3 | 4.9 | 23 | 0.79 | — | — |
| 11 | PDAD | 10.2 | 4.9 | 98 | 0.95 | — | — |
| 12 | PDSQ | 8.5 | 5.9 | 20 | 0.71 | — | — |
| 13 | PDSQ | 6.2 | 8.1 | 95 | 0.91 | — | — |
| 14 | PDAD | 5.6 | 8.9 | 58 | 0.85 | — | — |
| 15 | 2° Renal | 3.7 | 13.5 | 92 | 0.85 | 7.7 | 0.92 |
| 16 | 1° Clear | 2.9 | 17.2 | 73 | 0.76 | — | — |
| 17 | 2° Renal | 0.7 | 71.4 | 155 | 0.54 | 41.1 | 0.73 |

[a]DTpot and ∅ in these columns have been calculated using $T_s$ values from the literature on corresponding cell types of bronchogenic carcinoma and not the standard assumed 15 h (see text).

Abbreviations used for histology are as in **Figure 1** but in addition SQ = Squamous carcinoma and AD = Adenocarcinoma.

**Figure 2** The distribution of Cell Loss (as a %) within histopathological groups of pulmonary tumours. Abbreviations are as before. The "y-axis" has been shortened.

**Figure 3** The relationship between Cell Loss (%) and % Thymidine Labelling index. The correlation coefficient between y and x is 0.6 ($P<0.0005$).

There is *no* apparent direct relationship between actual doubling time of the tumour and TLI of the tumour cell population (Table II).

The relationship between cell loss and thymidine labelling index is shown in Figure 3. This graph suggests that, as the TLI of a tumour cell population increases the degree of cell loss from that tumour also increases. Thus, there appears to be a tendency for tumours with TLI > 17% to have cell loss factors of 90% or more whereas tumours with TLI < 5% have cell losses of < 90%.

### Discussion

### *Obtaining actual doubling time*

Between 8 and 31% of primary bronchogenic carcinomata may be "measurable" for the purposes of determining growth rate (Chahinian & Israel, 1976). Twenty-six percent of our cases on which TLI data were available were considered suitable for determination of DT. All these cases were surgically "operable" on clinical staging. The use of a surgical series may have selected out cases early in their natural history and with peripheral rather than hilar lesions more likely to be measurable.

### *Calculating potential doubling time and cell loss factor*

Estimation of DTpot for a tumour cell population from a knowledge of the thymidine labelling index of the population is based on well established theoretical work (Steel, 1967, 1968). Published data for the length of the S-phase ($T_s$) in human tumours suggests that assuming $T_s$ to be 15h is reasonably justified for the cases in this study (Frindel *et al.*, 1968; Terz *et al.*, 1971; Muggia *et al.*, 1974; Straus & Moran, 1977; Rabes *et al.*, 1979). Comparative calculations of DTpot shown in Table II using both assumed and published $T_s$ values supports this. Apart from these assumptions, doubt may be cast on the calculated values of TLI. We have discussed elsewhere the *in vitro* [$^3$H]-TdR labelling technique and why our data are probably more accurate than many others on determination of TLI (Kerr *et al.*, 1983). In any case the errors estimating DTpot in this way are not even of the appropriate order of magnitude to account for the large differences (often a factor of between 10 and 100) between DTpot and DT.

### *Actual doubling time of lung tumours*

It is clear from the literature (see Table I) that, within those groups of tumour of differing histological type which have been adequately studied, there is a very wide range of DT. The values for DT in the various histological groups in this series are similar to those published elsewhere with one exception, our small group of adenocarcinomata. In this group the case with the shortest DT (23 days) was a well differentiated adenocarcinoma of broncho-alveolar type. This value is outwith the published range of values for this pattern of tumour, which is from 159 to 590 days (Weiss *et al.*, 1966; Meyer, 1973; Steele & Buell, 1973). This is a group of tumours with a wide range of growth rates and an unusual structure which does not fit the usual model of a solid neoplasm. The relationship of DT to degree of differentiation of tumours has been reviewed by Charbit *et al.* (1971) who suggested that, in general, well differentiated tumours tend to have longer DT than poorly or undifferentiated tumours. Our results are, in general, in keeping with this.

Although Malaise *et al.* (1973) produced some evidence in their review of published data that there was a negative correlation between tumour DT and TLI (as TLI increased, DT decreased) we cannot support this suggestion.

### *The "parallel study" cases*

We can find only one previous report in the literature of the concurrent study of actual tumour growth and cell kinetic parameters of human tumours. Bresciani *et al.* (1974) studied five squamous carcinomata of the skin, lip or gum. They obtained detailed kinetic data including measurements of $T_s$ and TLI on each tumour using an *in vivo* labelling technique involving intracarotid [$^3$H]-TdR infusion and multiple tumour biopsy. However, their actual growth measurements were made over only two to three weeks and involved caliper measurements of the cutaneous nodules. In two cases no growth was detected during the observation period but DT was estimated based on the maximum changes in size that could have occurred yet which remained undetectable by their method of tumour measurement by calipers. Cell loss factors were found ranging from 78%–93%. They found that the *rate* of cell production and the *rate* of cell loss (per hour) for the tumours was negatively correlated with DT. When they compared *cell loss factor* with DT, $\phi$ increased slightly as DT increased.

Using pooled data on a heterogeneous series of human tumours Steel (1972) showed that cell loss factor $\phi$ again increased as DT increased and conversely that tumours generally with DT < 100 days showed a lower $\phi$ value than those with DT > 100 days. This group of human tumours, however, contained both carcinomata and sarcomata and if one considers the carcinomata alone the change in $\phi$ with change in DT is less apparent. From our data comparing those tumours with DT > 80 days with those of DT < 80 days there is NO statistical difference between the groups.

In experimental animal tumours parallel studies have been performed (Frindel *et al.*, 1967) and a trend of increasing cell loss factor with increasing DT seems apparent (Steel, 1968).

A few other individual human tumours of various site and type have cell loss values reported in the literature (Frindel *et al.*, 1968; Shirakawa *et al.*, 1970; Terz *et al.*, 1971). These tumours had detailed cell kinetic analysis made on them but DT was *not* concurrently measured. Malaise *et al.* (1973) reviewed the literature and did some comparative calculations for a wide variety of human tumours. Adenocarcinomata and squamous cell carcinomata (both heterogeneous groups from various primary sites) showed values of 71% and 91% respectively for the groups. Unfortunately none of these data refer specifically to primary bronchogenic adeno- or squamous cell carcinomata so that comparison with our data is of limited value.

Our figures do reinforce the generally held view that cell loss from human carcinomata is high. Furthermore our data would indicate that, in some primary lung cancers, cell loss is very high indeed. Almost two thirds of our primary bronchogenic carcinomata showed cell loss of 90% or more. In some cases ∅ even exceeds the 98% figure Bone & Camplejohn (1973) suggested rectal adenocarcinoma might have. Although the individual histological groups have small numbers of cases it seems that cell loss is higher in undifferentiated carcinomata than in the other histological groups.

As mean cell loss increases from groups of tumours of one histological type to another then so does the mean TLI of the group. Thus as growth fraction increases cell loss does also (Malaise *et al.*, 1973; Tubiana & Malaise, 1975). Our data confirm the relation between cell loss increase and both increasing % TLI and loss of tumour differentiation. Why does this relationship exist? Is it that cell replication carries with it a certain "casualty rate" or that increased expansion rate in a cell population means too great a demand on nutrient supply leading to cell death (Malaise *et al.*, 1973)? Of relevance in this context is the finding that increased cell loss makes tumour response to chemotherapy more likely (Bagshawe, 1968); this being especially so, as we might expect, in tumours with a high TLI (Tubiana & Malaise, 1975). How do these observations fit with the general clinical experience with chemotherapy in bronchogenic carcinoma? Chemosensitivity appears confined to a group of small cell undifferentiated carcinomata yet our data suggest that ∅ may be just as high, or even higher in large cell undifferentiated or squamous cell carcinomata. In addition small cell undifferentiated carcinomata appear to have, as a group, shorter DT than other primary lung cancers while mean TLI for this group is probably similar (Kerr *et al.*, 1983) to other groups'. Thus mean ∅ in small cell cancer as a group may be less than in other types of bronchogenic carcinoma. Adequate data are sparse but it is possible that more detailed investigation may reveal subpopulations of small cell carcinoma with "kinetic" differences.

This parallel study of actual growth and simple cell kinetic parameters has shown that cell loss from the tumour cell population is an important determinant of actual growth rate in human pulmonary neoplasms. Other important determinants are growth fraction and cell cycle time. In human tumours *ALL* these factors, however, are difficult to measure. While we are very aware of the errors involved with, and the criticisms that have been levelled against the measurements, assumptions and calculations we have been concerned with in this study (Garland *et al.*, 1963; Gurland & Johnson, 1965, 1966; Steel, 1968; Steel and Lamerton, 1969) data such as these may help towards a better understanding of the behaviour of human bronchogenic carcinoma.

Part of this work was supported by a grant from the National Coal Board and from the Scottish Hospital Endowment Research Trust to Dr D. Lamb. We thank: The late Mr R. McCormack, Mr P. Walbaum and Mr E. Cameron, Consultant Surgeons at the Thoracic Unit, City Hospital, Edinburgh, their colleagues, and the nursing and theatre staff for the supply of fresh lung resection specimens; Mr R. Hogg and Mr S. McKenzie for technical assistance; Mr A. McLean for assistance and advice regarding statistical analysis of data; Mrs Fiona Govan for typing this manuscript.

**References**

BAGSHAWE, K.D. (1968). Tumour growth and antimitotic action. The role of spontaneous cell losses. *Br. J. Cancer*, **22**, 698.

BONE, G. & CAMPLEJOHN, R. (1973). The role of cellular immunity in control of neoplasia. *Br. J. Surg.*, **60**, 824.

BRESCIANI, F., PAOLUZI, R., BENASSI, M., NERVI, C., CASALE, C. & ZIPARO, E. (1974). Cell kinetics and growth of squamous cell carcinomas in man. *Cancer Res.*, **34**, 2405.

CHAHINIAN, A.P. (1972). Relationship between tumour doubling time and anatomoclinical features in 50 measurable pulmonary cancers. *Chest*, **61**, 340.

CHAHINIAN, A.P. & ISRAEL, L. (1976). Rates and patterns of growth in lung cancer. In: *Lung Cancer: Natural History, Prognosis and Therapy*. (Eds. Israel & Chahinian). New York: Academic Press. p. 00

CHARBIT, A., MALAISE, E.P. & TUBIANA, M. (1971). Relation between the pathological nature and the growth rate of human tumours. *Eur. J. Cancer*, **7**, 307.

CLEAVER, J.E. (1967). *Thymidine Metabolism and Cell Kinetics*. Amsterdam, North Holland.

COLLINS, V.P., LEOFFLER, R.K. & TIVEY, H. (1956). Observations on growth rates of human tumours. *Am. J. Roentgenol.*, **76**, 988.

FRINDEL, E., MALAISE, E.P., ALPEN, E. & TUBIANA, M. (1967). Kinetics of cell proliferation of an experimental tumour. *Cancer Res.*, **27**, 1122.

FRINDEL, E., MALAISE, E.P. & TUBIANA, M. (1968). Cell proliferation kinetics in 5 human solid tumours. *Cancer*, **22**, 611.

GARLAND, L.H., COULSON, W. & WOLLIN, E. (1963). The rate of growth and apparent duration of untreated primary bronchial cancer. *Cancer*, **16**, 694.

GURLAND, J. & JOHNSON, R.O. (1965). How reliable are tumour measurements? *J.A.M.A.*, **194**, 125.

GURLAND, J. & JOHNSON, R.O. (1966). Case for using only maximum diameter in measuring tumours. *Cancer Chemother. Rep.*, **50**, 119.

ISRAEL, L., CHAHINIAN, P., ACCARD, J.L. & 7 others and the members of the measurable tumours group of the E.O.R.T.C. (1973). Growth curve modification of measurable tumours by 75 mg/m$^2$ of CCNU every 3 weeks. *Eur. J. Cancer*, **9**, 789.

KERR, K.M., ROBERTSON, A.M.G. & LAMB, D. (1983). In vitro thymidine labelling of human pulmonary neoplasms. *Br. J. Cancer*, **47**, 245.

MALAISE, E.P., CHAVAUDRA, N. & TUBIANA, M. (1973). The relationship between growth rate, labelling index and histological type of human tumour. *Eur. J. Cancer*, **9**, 305.

MEYER, J.A. (1973). Growth rate vs. prognosis in resected primary bronchogenic carcinoma. *Cancer*, **31**, 1468.

MUGGIA, F.M., KREZOSKI, S.K. & HANSEN, H.H. (1974). Cell kinetic studies in patients with small cell carcinoma of the lung. *Cancer*, **34**, 1683.

QUASTLER, H. & SHERMAN, F.G. (1959). Cell population kinetics in the intestinal epithelium of the mouse. *Exp. Cell. Res.*, **17**, 20.

RABES, H.M., CARL, P., MEISTER, P. & RATTENHUBER, U. (1979). Analysis of proliferative compartments in human tumours. I. Renal adenocarcinoma. *Cancer*, **44**, 799.

SCHWARTZ, M. (1961). A biomathematical approach to clinical tumour growth. *Cancer*, **14**, 1274.

SHIRAKAWA, S., LUCE, J.K., TANNOCK, I.F. & FREI, E. III. (1970). Cell proliferation in human melanomas. *J. Clin. Invest.*, **49**, 1188.

SPRATT, J.S. (Jr), SPJUT, H.J. & ROPER, C.L. (1963). The frequency distribution of the rates of growth and the estimated duration of primary pulmonary carcinomas. *Cancer* **16**, 687.

SPRATT, J.S. (Jr), & SPRATT, T.C. (1964). Rates of growth of pulmonary metastases and host survival. *Ann. Surg.*, **159**, 161.

STEEL, G.G. (1967). Cell loss as a factor in the growth rate of human tumours. *Eur. J. Cancer*, **3**, 381.

STEEL, G.G. (1968). Cell loss from experimental tumours. *Cell Tissue Kinet.*, **1**, 193.

STEEL, G.G. (1972). The cell cycle in tumours: An examination of data gained by the technique of labelled mitosis. *Cell Tissue Kinet.*, **5**, 87.

STEEL, G.G. (1977). Cell population kinetics in relation to the growth and treatment of cancer. In: *Growth Kinetics of Tumours*. Oxford: Clarendon Press.

STEEL, G.G. & LAMERTON, L.F. (1969). Human tumour cell kinetics. *Natl Cancer Inst. Monogr.*, **30**, 29.

STEELE, J.D. & BUELL, P. (1973). Asymptomatic pulmonary nodules. Host survival, tumour size and growth rate. *J. Thor. Cardiovasc. Surg.*, **65**, 140.

STRAUS, M.J. & MORAN, R.E. (1977). Cell cycle parameters in human solid tumours. *Cancer*, **40**, 1453.

TERZ, J.J., CURUTCHET, H.P. & LAWRENCE, W. (1971). Analysis of the cell kinetics of human solid tumours. *Cancer*, **28**, 1100.

TUBIANA, M. & MALAISE, E.P. (1975). Growth rate and cell kinetics in human tumours. Some prognostic and therapeutic implications. In: *Scientific Foundations of Oncology* (Eds. Symington & Carter) London, Heinmann.

WEISS, W., BOUCOT, K.R. & COOPER, D.A. (1966). Growth rate in the detection and prognosis of bronchogenic carcinoma. *J.A.M.A.*, **198**, 1246.